# The ability of *sarA* to limit protease production plays a key role in the pathogenesis of *Staphylococcus aureus* osteomyelitis irrespective of the functional status of *agr*

**DOI:** 10.1128/iai.00473-24

**Published:** 2024-11-29

**Authors:** Karen E. Beenken, Mara J. Campbell, Mark S. Smeltzer

**Affiliations:** 1 Department of Microbiology and Immunology, University of Arkansas for Medical Sciences 318223 , Little Rock, Arkansas, USA; 2 Department of Orthopaedic Surgery, University of Arkansas for Medical Sciences 414812 , Little Rock, Arkansas, USA; St Jude Children's Research Hospital, Memphis, Tennessee, USA

**Keywords:** *sarA*, agr, *Staphylococcus aureus*, osteomyelitis, proteases, pathogenesis

## Abstract

We demonstrate that mutation of the staphylococcal accessory regulator A (*sarA*) in the USA300 strain LAC limits virulence in a murine osteomyelitis model to a greater extent than mutation of the accessory gene regulator (*agr*) and that it does so irrespective of the functional status of *agr*. Protease production was decreased in the *agr* mutant but increased in *sarA* and *sarA/agr* mutants to a degree that limited biofilm formation. Extracellular protein A (eSpa) and full-length extracellular nuclease (Nuc1) were absent in the conditioned medium (CM) from *sarA* and *sarA*/*agr* mutants, and their abundance was restored in both mutants by eliminating protease production. Cytotoxicity of CM for osteoblasts and osteoclasts was also reduced in both mutants. Cytotoxicity was restored in a protease-deficient *sarA* mutant but not in the protease-deficient *sarA*/*agr* mutant. Reduced cytotoxicity was correlated with the reduced abundance of full-length α-toxin, LukF, and LukS in *sarA* and *sarA*/*agr* mutants. The abundance of these toxins in their full-length form was increased in the protease-deficient *sarA* mutant by comparison to LAC, demonstrating that mutation of *sarA* increases the production of these toxins but increased protease production limits their abundance in full-length and presumably functional forms. Most importantly, eliminating protease production enhanced the virulence of *sarA* and *sarA/agr* mutants, but had no impact in the *agr* mutant. We conclude that a key factor in the attenuation of LAC *sarA* and *sarA*/*agr* mutants in osteomyelitis is the increased production of extracellular proteases and its impact on virulence factors that contribute to biofilm formation and cytotoxicity.

**IMPORTANCE:**

The persistent emergence of antibiotic-resistant strains has rekindled interest in anti-virulence strategies to combat *S. aureus* infections. Numerous reports describe anti-virulence strategies focusing on key regulatory elements that globally influence virulence factor production, the two most commonly targeted being the accessory gene regulator (*agr*) and the staphylococcal accessory regulator A (*sarA*). We demonstrate that mutation of *sarA* limits virulence to a greater extent than mutation of *agr* and that this can be attributed to increased protease production in both *sarA* and *sarA*/*agr* mutants. This illustrates the critical role of *sarA* in protease-mediated post-translational regulation in *S. aureus*. It also suggests that an inhibitor of *sarA* would be more effective than an inhibitor of *agr* in overcoming the therapeutic recalcitrance of osteomyelitis and that such an inhibitor would remain effective even in the context of *agr* mutants known to arise *in vivo* during the transition from acute to chronic infection.

## INTRODUCTION

*Staphylococcus aureus* employs a complex and highly integrated regulatory system to control the production of its arsenal of virulence factors (1, 2). Two of the best studied regulatory loci at the center of this system are the accessory gene regulator (*agr*) and the staphylococcal accessory regulator (*sarA*). The agr system is a quorum-sensing system that modulates the production of AgrA and a regulatory RNA designated RNAIII, the accumulation of which defines a transition from the production of surface-associated virulence factors to the production of extracellular toxins and enzymes. This transition is evident *in vitro* as cultures go from the exponential to stationary growth phases (2). The effector molecule of the *sarA* system is a 15-kDa protein (SarA) that modulates the transcription of many *S. aureus* genes, including *agr* itself, with SarA being required for maximum expression of *agr* (1, 3).

Mutation of *sarA* results in increased protease production and decreased virulence in murine models of sepsis and osteomyelitis, and eliminating the ability of *sarA* mutants to produce extracellular proteases restores virulence to a statistically significant extent (4–9). Mutation of *agr* has the opposite effect on protease production but also limits virulence in these models (9, 10). This suggests that the impact of mutating *agr* on virulence is mediated at the level of protein production, while that of *sarA* is mediated at the level of protein degradation. It also suggests that the impact of mutating *sarA* on virulence is independent of *agr*. Nevertheless, the relative impact of *agr* and *sarA* on the pathogenesis of osteomyelitis remains unknown, particularly given that *sarA* is required for maximum expression of *agr* (1, 3, 11, 12). However, we are unaware of any studies that have made direct comparisons necessary to definitively assess the relative impact of the *agr*-dependent and *agr*-independent pathways of *sarA*-mediated regulation in the pathogenesis of osteomyelitis.

It is important to address this issue because *sarA* and *agr* are the two regulatory loci that have been evaluated most extensively as targets for prophylactic and therapeutic intervention in *S. aureus* infection (12–19). Spontaneous *agr* mutants arise *in vivo* and ultimately become the dominant subpopulation, and it has been proposed that this promotes the transition from acute to chronic infection (18–22). This suggests that inhibitors of *agr* might have the unintended consequence of promoting this transition. If the impact of *sarA* is *agr*-dependent, then inhibitors of *sarA* might have the same effect, while if the impact of *sarA* is *agr*-independent, this presumably would not be the case. Additionally, an effective inhibitor of *sarA* could retain its efficacy even in the context of these *agr* mutants.

In this report, we addressed the relative impact of *sarA* and *agr* on the pathogenesis of osteomyelitis by generating isogenic *sarA*, *agr*, and *sarA*/*agr* mutants in the USA300 strain LAC and assessing their relative virulence in a murine osteomyelitis model. To assess the role of extracellular proteases, we also evaluated derivatives of LAC and each regulatory mutant unable to produce the extracellular proteases aureolysin (Aur), staphopain A (ScpA), serine protease A (V8 protease, SspA), cysteine protease B (staphopain B, SspB), and the serine protease-like proteases (SplA-F). These 10 proteases were chosen because they are the primary *S. aureus* extracellular proteases and because eliminating their production was previously shown to impact virulence in LAC and its isogenic *sarA* mutant (4–7, 23).

## RESULTS

### Relative impact of *sarA* and *agr* on the pathogenesis of osteomyelitis

**Fig 1:**
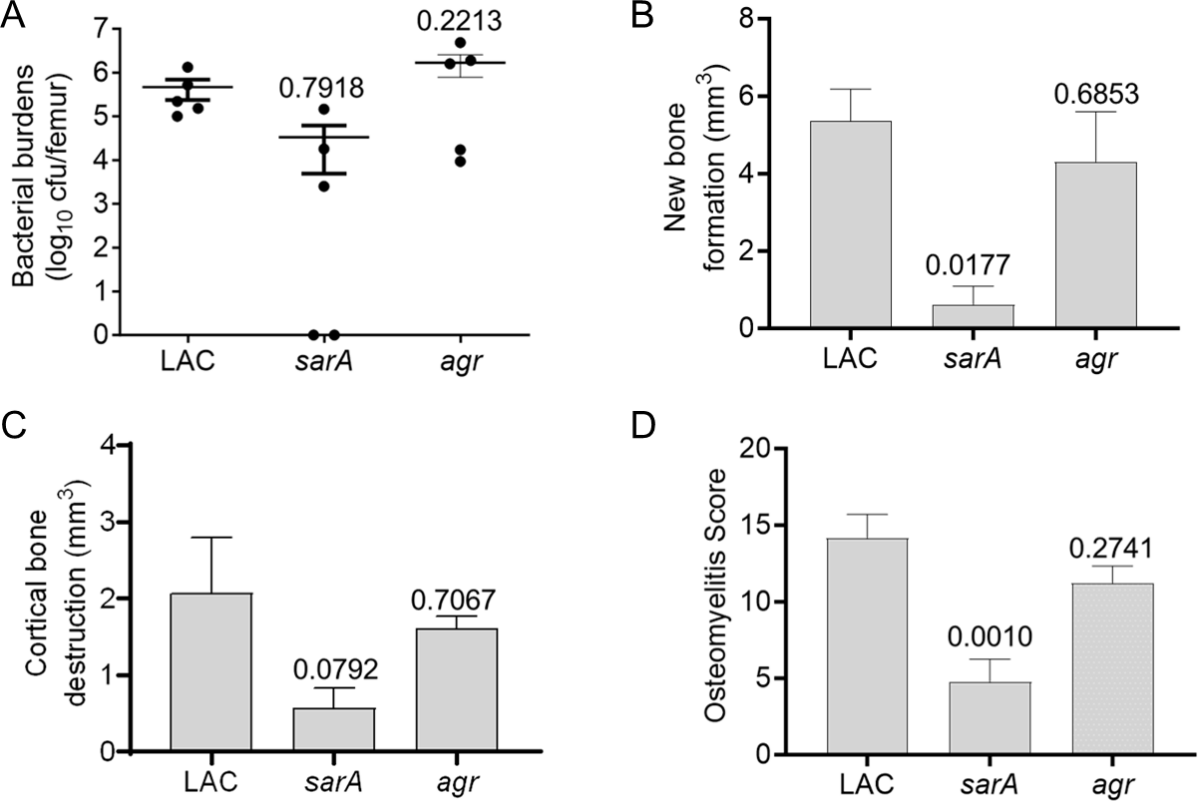
Impact of *sarA* and *agr* on virulence in a murine osteomyelitis model. Virulence in a murine osteomyelitis was assessed based on bacterial burdens in the femur (**A**), reactive new bone formation (**B**), cortical bone destruction (**C**), and cumulative osteomyelitis score (**D**). Numbers indicate *P* values based on one-way ANOVA comparing the results observed with LAC to those observed with isogenic *sarA* and *agr* mutants.

We used an established murine osteomyelitis model (4, 5) to make direct comparisons between mice infected with LAC and its isogenic *sarA* and *agr* mutants. We did not observe a statistically significant difference in the number of bacteria isolated from the femurs of mice infected with the *sarA* or *agr* mutants, but a downward trend was observed in mice infected with the *sarA* mutant (*P* = 0.7918), while an upward trend was observed in mice infected with the *agr* mutant (*P* = 0.2213) (Fig. 1A).

As assessed by quantitative micro-computed tomography, reactive new bone formation (NBF) was significantly reduced in mice infected with the *sarA* mutant (*P* = 0.0177) by comparison to mice infected with LAC (Fig. 1B). There was also a reduction in the NBF in mice infected with the *agr* mutant, but it was not statistically significant (*P* = 0.6853). Although the differences were not statistically significant, mice infected with *sarA* and *agr* mutants exhibited less cortical bone destruction (CBD), and like NBF, the decrease was greater in mice infected with the *sarA* mutant (*P* = 0.0792) than mice infected with the *agr* mutant (*P* = 0.7067) (Fig. 1C). Combining the results from all of these parameters into a cumulative osteomyelitis score (4, 5), it was revealed that mice infected with the *sarA* mutant exhibited a significant reduction in virulence (*P* = 0.0010), while mice infected with the *agr* mutant did not (*P* = 0.2741) (Fig. 1D). Based on these results and the possibility that the differences we observed might be related to protease production, we generated a *sarA*/*agr* mutant and derivatives of LAC and its regulatory mutants with null mutations in the genes and operons encoding aureolysin, ScpA (staphopain A), SspA, SspB (staphopain B), and the serine protease-like proteases SplA-F (4–9, 24).

### Impact of *sarA* and *agr* on protease activity and biofilm formation

**Fig 2:**
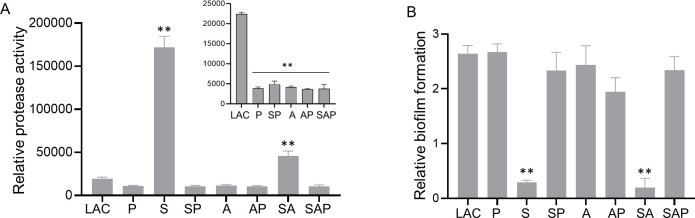
Impact of *sarA* and *agr* on protease production and biofilm formation. Protease production (**A**) and biofilm formation (**B**) were assessed in LAC, isogenic derivatives with mutations in *sarA* (S), *agr* (A), *sarA* and *agr* (SA), and protease-deficient derivatives of LAC and its isogenic sarA, *agr*, and *sarA/agr* mutants that do not produce aureolysin (Aur), staphopain A (ScpA), serine protease A (V8 protease, SspA), cysteine protease B (staphopain B, SspB), or the serine protease-like proteases (SplA-F). (P, SP, AP, and SAP, respectively). Asterisks indicate a significant increase in protease production (**A**) or decrease in biofilm formation, (**B**) as determined by one-way ANOVA with Dunnett’s correction for comparison to the results observed with LAC. The difference in protease production between the *sarA* and *sarA/agr* mutants was also significant (*P* < 0.0001), while the difference in biofilm formation was not. The inset in panel A shows data from the same experiment after excluding the *sarA* and *sarA*/*agr* mutants. Asterisks indicate a significant decrease in protease production, relative to LAC.

There was a significant reduction in the overall protease activity in all protease-deficient strains and a comparable reduction in the isogenic *agr* mutant (Fig. 2A, inset). There was a significant increase in protease activity in the *sarA* and *sarA*/*agr* mutants, although the increase was greater in the *sarA* mutant (Fig. 2A). However, the increase in the *sarA*/*agr* mutant was sufficient to limit biofilm formation to a comparable degree, and in both mutants, biofilm formation was restored by eliminating protease production (Fig. 2B). Mutation of *agr* did not have a significant impact on biofilm formation, irrespective of protease production (Fig. 2).

### Impact of *sarA* and *agr* on the extracellular proteome

**Fig 3:**
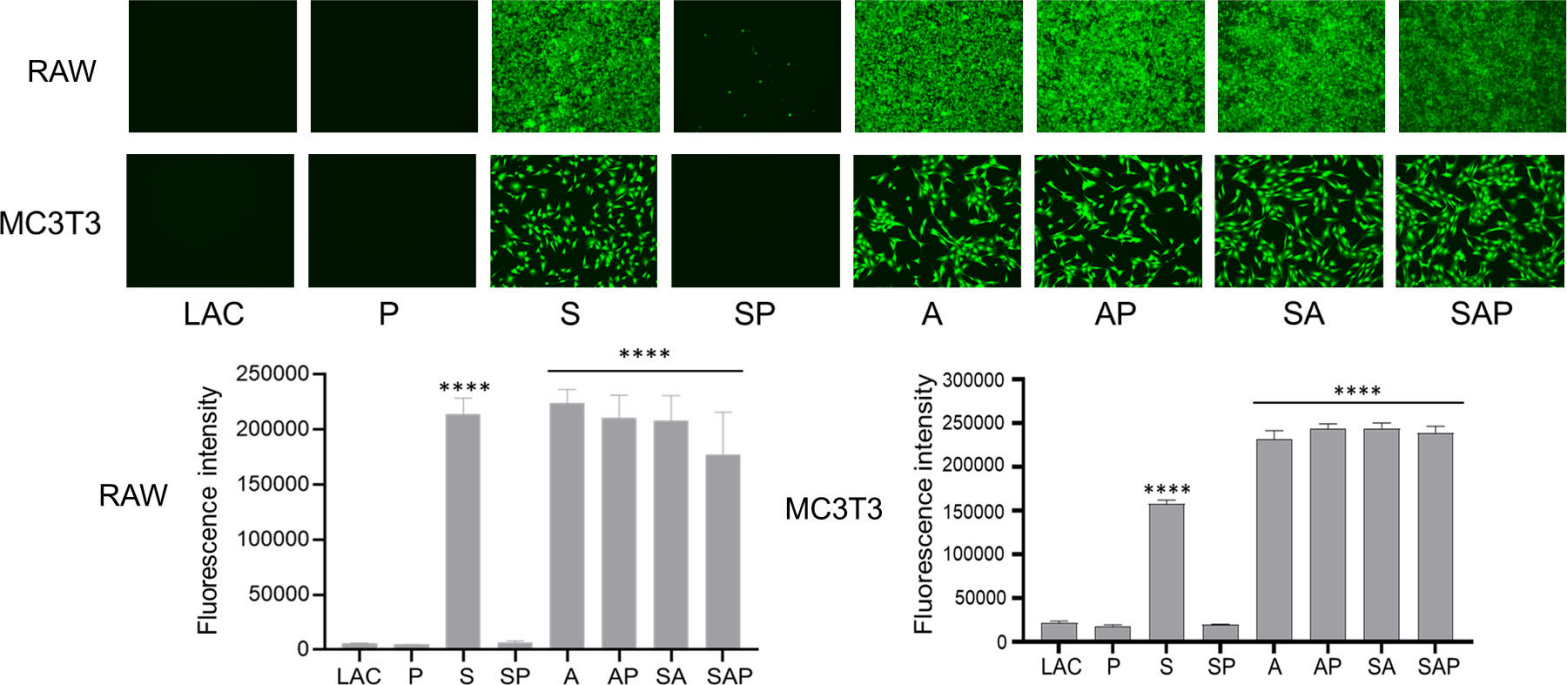
Impact of *sarA* and *agr* on cytotoxicity as a function of protease activity. RAW and MC3T3 cells were used for surrogates for osteoclasts and osteoblasts, respectively. Cytotoxicity was assessed using CM from LAC; isogenic derivatives with mutations in *sarA* (S), *agr* (A), and *sarA* and *agr* (SA); and protease-deficient derivatives of all four strains (P, SP, AP, and SAP, respectively). Asterisks indicate a statistically significant difference by comparison to the results observed with LAC. Results are reported as the fluorescence intensity, with increased fluorescence reflecting increased cell viability.

SDS-PAGE analysis demonstrated the absence of high-molecular weight proteins (>40 kDa) in conditioned media from *sarA*, *agr*, and *sarA*/*agr* mutants (Fig. S1). These changes were correlated with reduced cytotoxicity for osteoclasts and osteoblasts. Specifically, CM from LAC, its protease-deficient derivative, and the protease-deficient *sarA* mutant were cytotoxic for both osteoblasts and osteoclasts (Fig. 3), while cytotoxicity was reduced in CM from all mutants in which the abundance of high-molecular weight proteins was limited (Fig. S1).

### Impact of *sarA* and *agr* on virulence factors implicated in cytotoxicity

**Fig 4:**
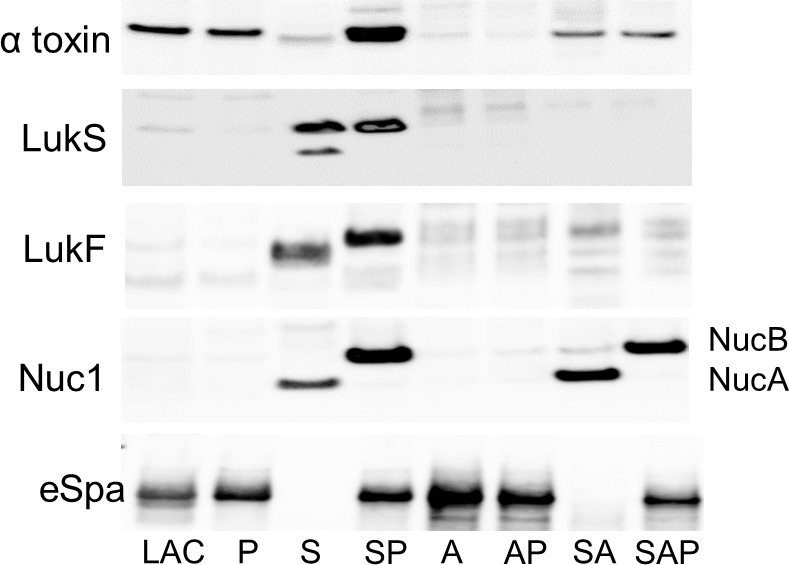
*sarA* limits the production of specific virulence factors via protease-dependent and -independent mechanisms. Western blots were done with CM from overnight cultures of LAC; isogenic derivatives with mutations in *sarA* (S), *agr* (A), and *sarA* and *agr* (SA); and protease-deficient derivatives of all of these strains (P, SP, AP, and SAP, respectively) using primary antibodies for protein A (Spa), α-toxin (Hla), leukocidin S (LukS), leukocidin F (LukF), and nuclease 1 (Nuc1). Nuc1 is proteolytically processed from its full-length form (NucB) into a truncated form (NucA).

By comparison to CM from LAC, α-toxin was diminished or absent in all mutants that exhibited reduced cytotoxicity (Fig. 4). The abundance of α-toxin was restored in CM from the protease-deficient *sarA* mutant and even increased by comparison to that in LAC and its protease-deficient derivative. This demonstrates that mutation of *sarA* results in the increased production of α-toxin and that its abundance is limited in the *sarA* mutant owing to increased protease production. Full-length α-toxin was not detectable in CM from the *agr* mutant and its protease-deficient derivative, but it was detected in CM from the *sarA*/*agr* mutant and its protease-deficient derivative (Fig. 4). CM from these strains was not cytotoxic (Fig. 3), suggesting that the amount of α-toxins in CM from *sarA*/*agr* mutants is below a critical threshold for our cytotoxicity assay.

LukS and LukF, the two components of the Panton–Valentine leukocidin (PVL), were not readily detectable by Western blot of CM from LAC or its protease-deficient derivative (Fig. 4), but CM from both strains was cytotoxic (Fig. 3). In contrast, LukF and LukS were detected in CM from the *sarA* mutant despite its reduced cytotoxicity. However, CM from the *sarA* mutant contained truncated forms of both proteins, and in the case of LukF, this was the only form detected (Fig. 4). Only full-length forms of LukF and LukS were detected in CM from the protease-deficient *sarA* mutant, which was cytotoxic (Fig. 3). The fact that LukF was only present in a truncated form in CM from the *sarA* mutant suggests that this could be a limiting factor in PVL-associated cytotoxicity.

Whether in truncated or full-length forms, the abundance of LukF and LukS was also increased in CM from the *sarA* and protease-deficient *sarA* mutants, thus demonstrating that mutation of *sarA* also results in the increased production of LukF and LukS (Fig. 4). In fact, mutation of *sarA* resulted in a much greater increase in the abundance of LukF and LukS than mutation of *sarS* (Fig. S2), which was previously implicated as a key repressor of leukocidin production in *S. aureus* (25).

### Impact of *sarA* and *agr* on virulence factors implicated in biofilm formation

The extracellular nuclease Nuc1 was also more abundant in CM from the *sarA* and protease-deficient *sarA* mutants (Fig. 4). However, in CM from the *sarA* mutant, all of Nuc1 was in the smaller NucA form, while in CM from the protease-deficient *sarA* mutant, it was in the larger NucB form. Both NucA and NucB are enzymatically active (26), suggesting that this difference may not be phenotypically apparent. However, this pattern was fully replicated in the *sarA*/*agr* mutant and its protease-deficient derivative (Fig. 4), thus demonstrating that the impact of *sarA* on Nuc1 production is not dependent on the functional status of *agr*.

Staphylococcal protein A (Spa) is produced in both surface-anchored and extracellular forms, and both forms contribute to biofilm formation and function as activators of osteoclasts to a degree associated with increased bone destruction in osteomyelitis (27, 28). The experiments we report focused on protein abundance in CM and thus were limited to extracellular Spa (eSpa). Unlike LukF, LukS, and Nuc1, all of which were absent in CM from the LAC *agr* mutant and its protease-deficient derivative, the abundance of eSpa was increased in CM from the *agr* mutant and its protease-deficient derivative (Fig. 4). In contrast, eSpa was absent in CM from the *sarA* and *sarA/agr* mutants, and its abundance was restored to levels comparable to those of LAC in their protease-deficient derivatives (Fig. 4).

### Impact of protease production on the attenuation of *sarA* and *agr* mutants

To assess *in vivo* relevance of these results, we used our osteomyelitis model to evaluate the virulence of LAC; its *sarA*, *agr*, and *sarA*/*agr* mutants; and derivatives of all four strains unable to produce extracellular proteases. Representative µCT images illustrate extensive NBF and CBD in mice infected with LAC and its protease-deficient derivative (Fig. S3). They also suggest that, by comparison to *sarA* and *sarA/agr* mutants, the mutation of *agr* has little effect on either of these pathological parameters, irrespective of the ability to produce extracellular proteases. In contrast, both were limited in mice infected with the isogenic *sarA* and *sarA/agr* mutants, and both were enhanced in isogenic protease-deficient derivatives (Fig. S3).

**Fig 5:**
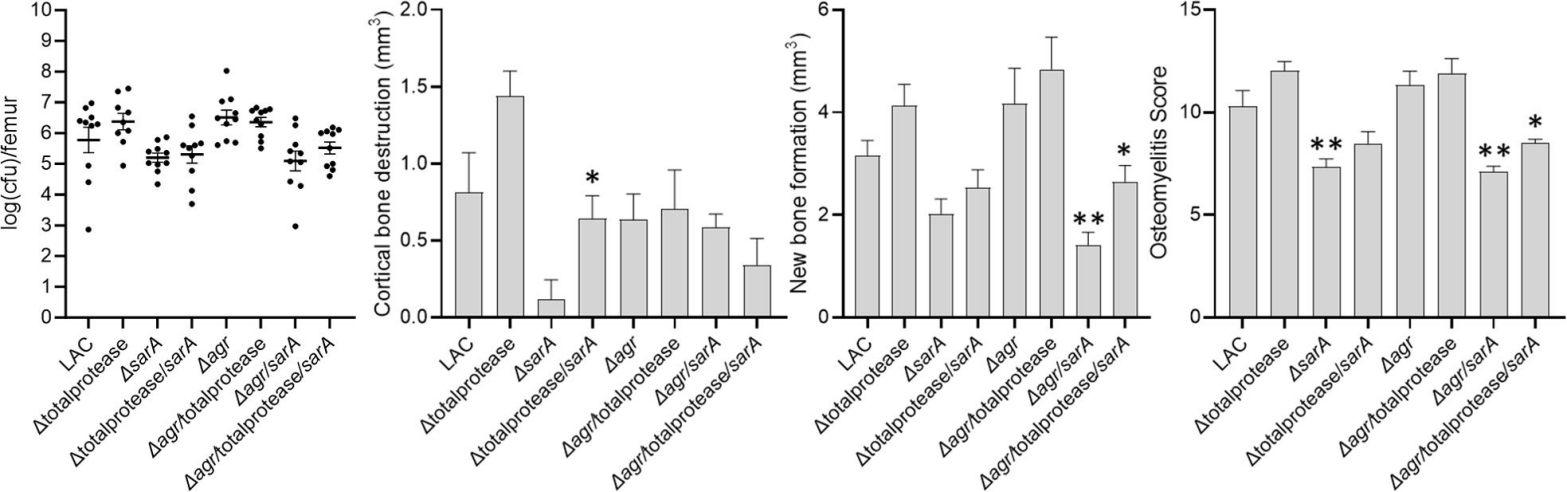
Quantitative analysis of infected femurs. Bacterial burdens in the femur, cortical bone destruction, new bone formation, and cumulative osteomyelitis score were determined for each mouse infected with LAC; its *sarA*, *agr*, and *sarA/agr* mutants; and protease-deficient derivatives of all four strains. Double asterisks indicate a statistically significant decrease (*P* ≤ 0.0439) by comparison to mice infected with LAC, as determined by one-way ANOVA. The reduction in cortical bone destruction in mice infected with the *sarA* mutant was not statistically significant (*P* = 0.0503). A single asterisk indicates a statistically significant increase in the protease-deficient derivative by comparison to its isogenic regulatory mutant, as determined by unpaired *t*-test.

These differences were confirmed by quantitative µCT analysis. Specifically, by comparison to mice infected with LAC, a reduction in CBD was observed in mice infected with the *sarA* mutant, although as assessed by one-way ANOVA, this difference was not statistically significant (*P* = 0.0503). Nevertheless, by comparison to mice infected with the *sarA* mutant, this was reversed to a statistically significant extent in mice infected with the protease-deficient *sarA* mutant (*P* = 0.0163) (Fig. 5). The only significant difference observed in mice infected with the *sarA*/*agr* mutant by comparison to mice infected with LAC was decreased NBF (*P* = 0.0243), and this was also reversed by eliminating the production of extracellular proteases (*P* = 0.0066). NBF was also reduced in mice infected with the *sarA* mutant by comparison to mice infected with LAC, but the difference was not statistically significant (*P* = 0.6429). NBF and CBD are both indicators of overall virulence in osteomyelitis, but this suggests a disconnect between NBF and CBD, which may reflect a spatial difference in osteoblast and osteoclast activity (29) that is driven by the functional status of *sarA* and *agr* relative to each other.

As in the preliminary experiment that provided the foundation for these expanded *in vivo* studies, no significant differences were observed in bacterial burdens in the femur, but downward trends were evident in mice infected with the *sarA* and *sarA/agr* mutants but not in mice infected with the *agr* mutant (Fig. 5). Moreover, when all of these virulence indicators were combined into a cumulative osteomyelitis score that also allows us to include fractured bones (4, 5), a statistically significant reduction in virulence was found in the *sarA* (*P* = 0.0021) and *sarA/agr* mutants (*P* = 0.0009), but not in the *agr* mutant. The impact of eliminating proteases was significant in the *sarA/agr* mutant (*P* = 0.0006), but not in the *sarA* mutant, although an upward trend was observed in the protease-deficient *sarA* mutant (*P* = 0.1139). In contrast, there were no significant differences between mice infected with *agr* and protease-deficient *agr* mutants, as assessed by the overall osteomyelitis score (*P* = 0.5682) or any of the individual indicators of virulence in our osteomyelitis model (Fig. 5).

Finally, bacterial burdens in the femur, cortical bone destruction, new bone formation, and overall osteomyelitis score were all increased in mice infected with the protease-deficient derivative of LAC itself, although none of these differences were statistically significant (Fig. 5). There was also definitive evidence of systemic dissemination from the bone to multiple soft tissues, which was limited to mice infected with the protease-deficient derivative of LAC (Fig. S4).

## DISCUSSION

The persistent problem of acquired antibiotic resistance in *S. aureus* has rekindled interest in the development of anti-virulence therapeutic strategies. Given the arsenal of *S. aureus* virulence factors, much of this effort has focused on key regulatory loci that control the abundance of multiple virulence factors. The staphylococcal accessory regulator (*sarA*) and accessory gene regulator (*agr*) are the two regulatory loci that have received the most interest in this regard (12–18). The interest in *agr* is based on its role in increasing production of *S. aureus* exotoxins and the role of these toxins in acute infections (30), while the interest in *sarA* is based on its role in biofilm formation and chronic infections (31). The fact that mutation of *sarA* limits biofilm formation while mutation of *agr* has the opposite effect largely defines this distinction (32, 33), although the impact of agr on biofilm formation has been called into question particularly under *in vivo* conditions (34). However, *agr*-defective variants of *S. aureus* are often isolated from patients, and it has been proposed that such mutants promote the transition between acute and chronic forms of *S. aureus* infection, including osteomyelitis (18–22). In contrast, we are unaware of any reports of *sarA* mutants or strains exhibiting altered protease activity being isolated from patients suffering from orthopedic infection or any other form of *S. aureus* infection.

Our clinical focus on biofilm-associated orthopedic infections accounts for our interest in *sarA* as a prophylactic and therapeutic target. Indeed, our previous studies confirmed that mutating *sarA* has a greater impact on protease production, biofilm formation, and virulence in osteomyelitis than mutation of any other *S. aureus* regulatory locus we have examined (4–9, 35–41). This is not to say that mutation of other regulatory loci does not have a significant effect on these phenotypes, one example being *saePQRS* (*sae*), but rather that mutation of these other loci has a reduced effect by comparison to *sarA*. However, our previous comparative studies were limited to *S. aureus* mutants that exhibit increased protease production, and as confirmed here, this does not include *agr*.

This accounts for our focus on *sarA* and *agr* in this report, and we believe the results we present confirm that mutation of *sarA* limits virulence in our osteomyelitis model to a greater extent than mutation of *agr*. As with *sae* (7), this is not to suggest that mutation of *agr* does not limit virulence in our osteomyelitis model. Indeed, we observed downward trends in mice infected with the LAC *agr* mutant, particularly with respect to CBD. However, the limited virulence observed in mice infected with an LAC *agr* mutant by comparison to mice infected with LAC was not statistically significant in either of our *in vivo* experiments. Rather, the collective results we report support the conclusion that mutation of *sarA* has a greater impact on the virulence of *S. aureus* in osteomyelitis than mutation of *agr* and that this is due to the increased production of extracellular proteases to a degree that is evident even in an *sarA*/*agr* mutant. This is important in the context of anti-virulence targets, in that our results suggest that an effective inhibitor of *sarA* would have a greater therapeutic effect than an inhibitor of *agr*, at least in the context of osteomyelitis, and that an *sarA* inhibitor would retain its efficacy even against the spontaneous *agr* mutants known to arise *in vivo* during the transition between acute and chronic *S. aureus* infections (18–22). Such inhibitors could be used along with conventional antibiotics as adjunct therapy following traumatic injury to the bone, perioperatively in elective orthopedic procedures including total joint arthroplasty or, given the frequent need for targeted local delivery of therapeutic agents following debridement and revision of infected bone and orthopedic implants, incorporated into local antibiotic delivery matrices (42, 43).

Our previous studies established that the increased production of proteases in *sarA* mutants occurs at a transcriptional level (6), and here we confirm that mutation of *sarA* also results in the increased production of specific virulence factors implicated in biofilm formation and cytotoxicity. This includes α-toxin, LukF, LukS, and Nuc1, all of which were present in increased amounts in CM from the protease-deficient *sarA* mutant by comparison to CM from LAC and its protease-deficient derivative. With the exception of α-toxin, which was undetectable in the *sarA* mutant, these proteins were also present in increased amounts in CM from the *sarA* mutant itself. In fact, the abundance of LukF and LukS was increased in CM from an LAC *sarA* mutant even above the levels observed in an LAC *sarS* mutant (Fig. S2), which was previously reported to play a primary role in limiting the production of *S. aureus* leukocidins (25). This demonstrates that *sarA* plays a critical role in limiting the production of α-toxin, LukF, and LukS and suggests that it does so in an *agr*-independent manner. However, we believe this would be an oversimplification. Specifically, these proteins were absent in CM from the LAC *agr* and isogenic *sarA*/*agr* mutants, thus demonstrating that the increased production of α-toxin, LukF, and LukS in the *sarA* mutant is dependent on the functional status of *agr*.

The α-toxin phenotype of the *sarA*/*agr* mutant provides a potential explanation that is independent of *agr* to the extent that it is not dependent on the influence of *sarA* on *agr* expression. Specifically, the α-toxin phenotype of the *sarA*/*agr* mutant was intermediate between that of the isogenic protease-deficient *sarA* mutant and the parent strain. We hypothesize that the abundance of α-toxin in the *sarA*/*agr* mutant may reflect increased transcription of the corresponding gene (*hla*) owing to the *sarA* mutation, but a limited capacity to produce α-toxin owing to the *agr* mutation and the absence of RNAIII, which is required for translation of *hla* mRNA (44). In this scenario, the observation that this intermediate phenotype was not apparent with LukF or LukS could reflect the importance of the balance between these two factors, thus further illustrating the complex and interactive nature of *sarA* and *agr*. Nevertheless, as suggested by the reduced cytotoxicity of the *sarA* mutant, the increased production of these proteins in an *sarA* mutant may be phenotypically irrelevant owing to the increased production of extracellular proteases and its impact on the abundance of full-length and presumably functional toxins.

It is noteworthy that mutation of *agr* limited the abundance of these same toxins, and this was correlated with reduced cytotoxicity for osteoblasts and osteoclasts. However, it was not correlated with reduced biofilm formation. In contrast, mutation of *sarA* limited both of these phenotypes to a degree that could be correlated with reduced virulence. Given the multifactorial nature of osteomyelitis and the potential importance of both of these *in vitro* phenotypes, this likely contributes to the reduced virulence of *sarA* mutants, irrespective of the functional status of *agr*. The increased production of extracellular proteases in both *sarA* and *sarA/agr* mutants, together with the fact that eliminating protease production enhanced the virulence of both mutants, is consistent with the conclusion that extracellular proteases play an important role in this regard.

This is consistent with the eSpa and Nuc1 phenotypes of *sarA* and *sarA*/*agr* mutants, both of which have been implicated in biofilm formation (26, 45). In fact, the observation that the phenotype of *sarA* mutants was precisely replicated in the isogenic *sarA/agr* mutant with both of these proteins further illustrates the importance of the *agr*-independent pathway of *sarA*-mediated regulation. In fact, mutation of *agr* resulted in an apparent increase in the abundance of eSpa as expected based on the current *agr* regulatory paradigm (2), and this was reversed in the protease-deficient *sarA* and *sarA*/*agr* mutants. The virtual absence of eSpa in CM from the *sarA* and *sarA*/*agr* mutants due to protease-mediated degradation may be particularly relevant, in that Spa contributes to osteomyelitis-associated phenotypes and the pathogenesis of osteomyelitis owing to its role in biofilm formation, osteoclastogenesis, and cortical bone destruction (27, 28).

Finally, *S. aureus* extracellular proteases are proven virulence factors that contribute to nutrient acquisition, tissue invasion, and avoiding host defenses (46–50). The results we present do not contradict this conclusion but rather demonstrate that the increased production of proteases contributes to the attenuation of *sarA* and *sarA*/*agr* mutants. To the extent that the inability to produce proteases enhances virulence in both mutants, our results are consistent with a scenario in which it is important for *S. aureus* to produce proteases for multiple reasons including balancing its virulence factor repertoire, but equally important that the production of these proteases is kept in check. Failing that, the increased production of extracellular proteases observed in *sarA* mutants limits the availability of multiple virulence factors that contribute to osteomyelitis-associated phenotypes, including biofilm formation and cytotoxicity for osteoblasts and osteoclasts, as evidenced by their impact on the virulence factors examined in this report. In fact, our results suggest that even in an *agr* mutant, the impact of extracellular proteases extends beyond their impact on these virulence factors to include other elements of the *S. aureus* proteome. The need to fine-tune protease production is further reflected in the number of *S. aureus* regulatory loci that have been implicated in this regard (6, 35). Thus, the results presented here confirm the critical importance of *sarA* in limiting protease production to levels that benefit *S. aureus* without compromising its own virulence factor repertoire and demonstrate that *sarA* is important in this regard irrespective of the functional status of *agr*.

## MATERIALS AND METHODS

### Bacterial strains and growth conditions

Mutants were generated in LAC by phage-mediated transduction, as previously described (35, 37, 39). Bacterial strains were recovered from frozen stock cultures and grown overnight (16 hr). Cultures of each strain were then standardized to an optical density (OD_560_) of 10.0. CM was prepared from each standardized culture by removing bacterial cells by centrifugation, followed by filter sterilization with 0.20-micron filters.

### Phenotypic assays

Total protease activity was assessed as previously described (4, 5) using CM from standardized bacterial cultures. Assays were done using the EnzChek Gelatinase/Collagenase Assay Kit (Thermo Fisher Scientific, Cat. #E12055). Biofilm assays were performed as previously described using plasma-coated microtiter plates and tryptic soy broth (TSB) supplemented with salt and glucose (32, 33). Cytotoxicity for osteoblasts and osteoclasts was assessed as previously described (4, 5) using RAW 264.7 and MC3T3-E1 cells as surrogates for osteoclasts and osteoblasts, respectively. All phenotypic assays were done with at least two biological replicates, each of which included at least three experimental replicates.

### SDS-PAGE and Western blot analysis

Protein electrophoresis of CM samples was done using 4%–12% gradient Bolt Bis-Tris Plus gels (Thermo Fisher Scientific). Gels were stained with SimplyBlue SafeStain (Thermo-Fischer Scientific) and imaged with a Bio-Rad ChemiDoc MP imaging system (Bio-Rad Laboratories) or used for Western blots. Western blots for extracellular protein A (eSpa), Nuc1, LukS, LukF, and α-toxin were done using commercially available antibodies (Sigma and Toxin Technologies, abCAM, and United States Biological) as previously described (4, 5).

### Murine osteomyelitis model

For *in vivo* experiments, each strain was grown overnight (16 hours) with constant shaking at 37°C in TSB without antibiotic selection. Bacterial cells were harvested by centrifugation, washed three times with sterile PBS, and resuspended in PBS at a density of 5 × 10^8^ colony-forming units (CFUs) per mL. Cell density and strain identity were confirmed by plating serial dilutions on TSA with and without antibiotic selection. Mice were infected with 1 × 10^6^ CFUs as previously described (4, 5). After 14 days, mice were humanely euthanized, and the femurs frozen at −80°C. Frozen femurs were used for μCT analysis, followed immediately by homogenization for determination of bacterial burdens. Soft tissues were also harvested immediately after euthanasia and homogenized to determine bacterial burdens in individual tissues. The identity of isolates obtained from bone and soft tissues was confirmed by plating on TSA with antibiotic selection for the appropriate mutations and by polymerase chain reaction (PCR) analysis to confirm the absence of *cna* (forward: CAAGCAGTTATTACACCAGACGG, reverse: CACCTTTTACAGTACCTTCAATACC) and the presence of *lukS* (forward: AATTGCATTGCTTTTGCTATCC, reverse: ATTTTGAACCATTACCTCCACC). Colony counts were logarithmically transformed for statistical analysis. Samples with no bacterial burden were assigned a colony count of 1 to allow inclusion of results from all experimental animals.

### Microcomputed tomography (μCT)

Femurs were scanned with a Skyscan 1275 Microtomograph (Bruker) at 40 kV (100 uA) using an isotropic voxel size of 6.8 um. After scanning, images were reconstructed using Skyscan Nrecon software and then processed with Skyscan CT-analyzer software as previously described (4–7). A semi-automated protocol (global thresholding 90–255, round closing in 3D with pixel size 4, round opening in 3D with pixel size 1, round closing in 3D with pixel size 8, and round dilation in 3D pixel size 3) was used to generate preliminary regions of interest (ROIs) of cortical bone. Every 20th automated ROI image was loaded onto the sample to act as a base for manual ROI adjustment to ensure inclusion of only cortical bone within the ROI. Cortical bone volume was then calculated within the ROIs with a threshold of 70–255, and cortical bone destruction was determined by subtracting the cortical bone volume from the average amount of cortical bone in a sham surgical bone. New bone ROIs were generated using the subtractive volume function on the cortical ROIs, calculating bone volume with a threshold of 45–135, and then subtracting the corresponding average amount of bone present in sham surgical bones to account for any trabecular bone inclusion as well as normal post-surgical new bone formation. Example uCT images from randomly selected bones from mice in each experimental group were generated in Skyscan CTvox software.

### Statistical analysis

Statistical analysis of *in vitro* and *in vivo* results was done by one-way ANOVA with Dunnett’s correction to allow comparison of the results from all experimental groups relative to the results observed with LAC. Unpaired *t*-tests were used to individually assess the impact of extracellular proteases in LAC and its regulatory mutants. Error bars indicate standard error of the mean. All statistical analyses were done using GraphPad Prism software (version 10.0.0 for Windows, GraphPad Software).

## Data Availability

The authors confirm that the data supporting the findings of this study are available within the article [and/or] its supplementary materials.

## SUPPLEMENTAL MATERIAL

Fig. S1 iai.00473-24-s0001.tif Supplemental Figure 1.

Fig. S2 iai.00473-24-s0002.tif Supplemental Figure 2.

Fig. S3 iai.00473-24-s0003.tif Supplemental Figure 3.

Fig. S4 iai.00473-24-s0004.tif Supplemental Figure 4.

ASM does not own the copyrights to Supplemental Material that may be linked to, or accessed through, an article. The authors have granted ASM a non-exclusive, world-wide license to publish the Supplemental Material files. Please contact the corresponding author directly for reuse.
