# Supplementary figures and images for: The ability of sarA to limit protease production plays a key role in the pathogenesis of Staphylococcus aureus osteomyelitis irrespective of the functional status of agr
Source: Infect Immun. 2024 Nov 29;93(1):e00473-24. doi: 10.1128/iai.00473-24 (PMC11784413; doi:10.1128/iai.00473-24)

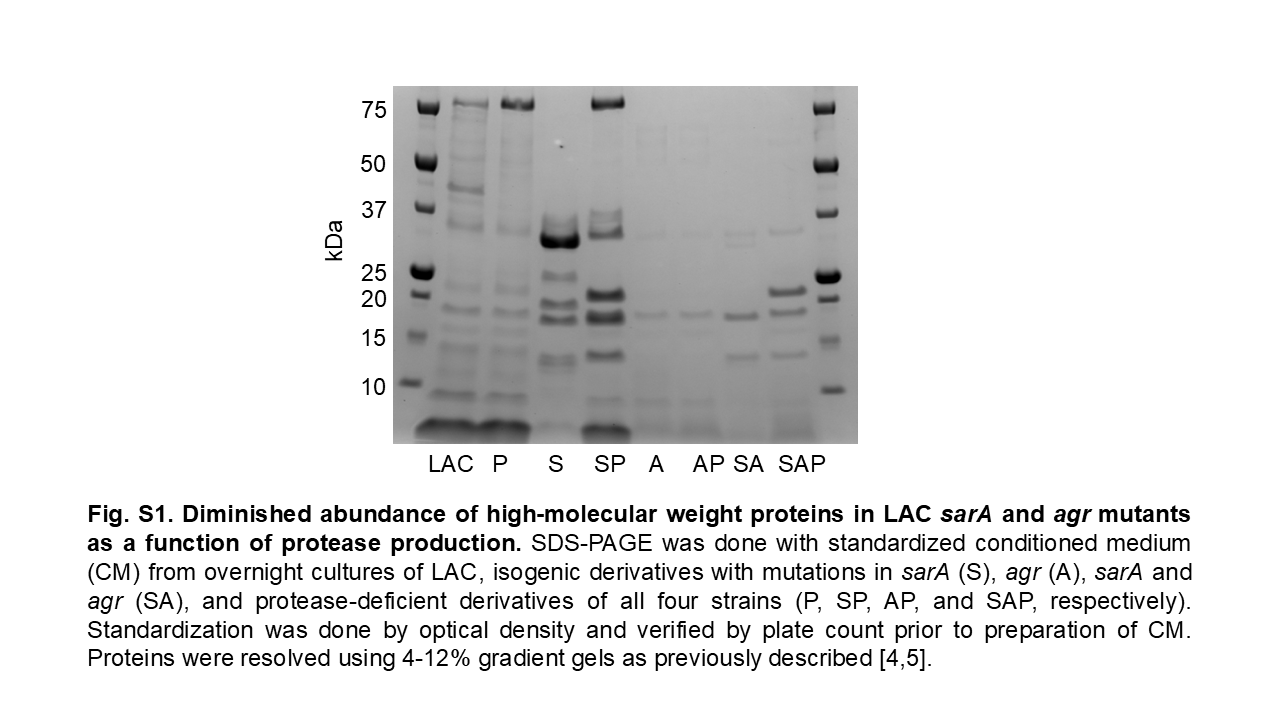

Supplement: Fig. S1 — Supplemental Figure 1. [file iai.00473-24-s0001.tif]

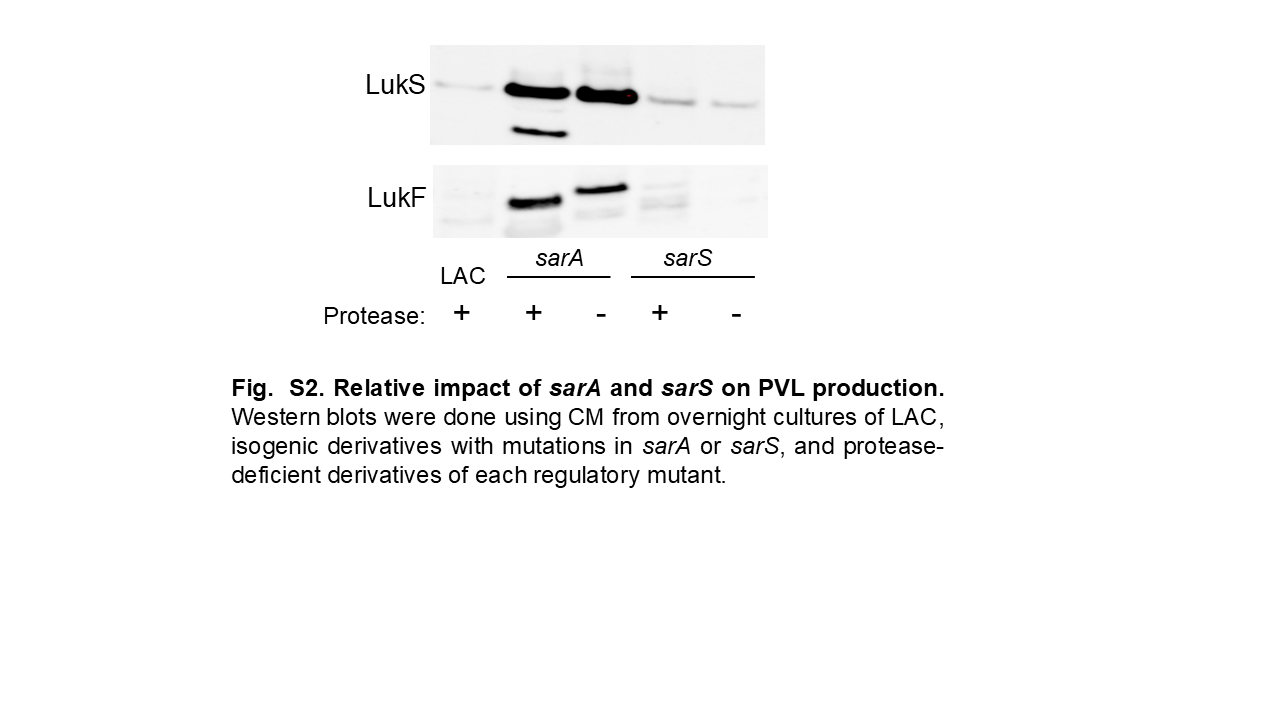

Supplement: Fig. S2 — Supplemental Figure 2. [file iai.00473-24-s0002.tif]

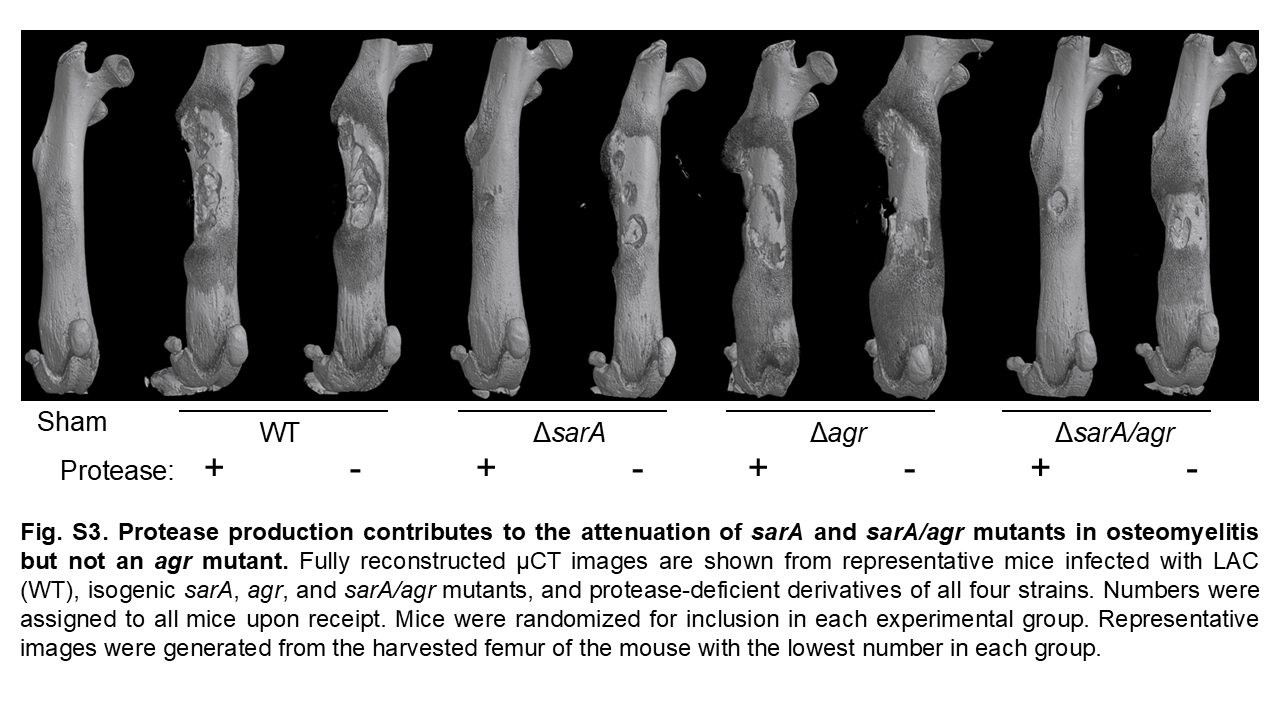

Supplement: Fig. S3 — Supplemental Figure 3. [file iai.00473-24-s0003.tif]

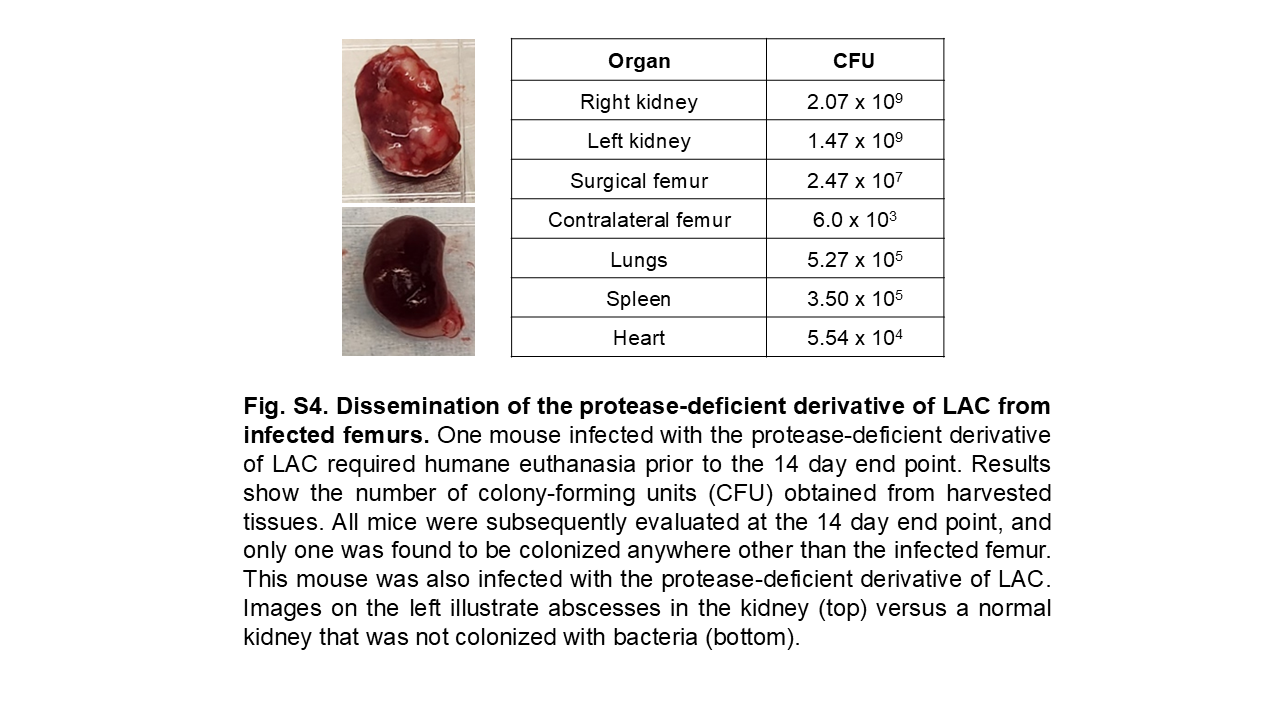

Supplement: Fig. S4 — Supplemental Figure 4. [file iai.00473-24-s0004.tif]
